# Assessing the Effects of Mild Traumatic Brain Injury on Vestibular Home Exercise Performance with Wearable Sensors

**DOI:** 10.3390/s23249860

**Published:** 2023-12-16

**Authors:** Kody R. Campbell, Jennifer L. Wilhelm, Prokopios Antonellis, Kathleen T. Scanlan, Natalie C. Pettigrew, Douglas N. Martini, James C. Chesnutt, Laurie A. King

**Affiliations:** 1 Department of Neurology, Oregon Health & Science University, Portland, OR 97239, USA; wilhelmj@ohsu.edu (J.L.W.); antonelp@ohsu.edu (P.A.); kingla@ohsu.edu (L.A.K.); 2 Department of Kinesiology, University of Massachusetts Amherst, Amherst, MA 01060, USA; 3 Department of Family Medicine, Oregon Health & Science University, Portland, OR 97239, USA

**Keywords:** wearable sensors, IMU, monitoring, vestibular, rehabilitation, concussion, balance, gait, mild traumatic brain injury

## Abstract

After a mild traumatic brain injury (mTBI), dizziness and balance problems are frequently reported, affecting individuals’ daily lives and functioning. Vestibular rehabilitation is a standard treatment approach for addressing these issues, but its efficacy in this population remains inconclusive. A potential reason for suboptimal outcomes is the lack of objective monitoring of exercise performance, which is crucial for therapeutic success. This study utilized wearable inertial measurement units (IMUs) to quantify exercise performance in individuals with mTBI during home-based vestibular rehabilitation exercises. Seventy-three people with mTBI and fifty healthy controls were enrolled. Vestibular exercises were performed, and IMUs measured forehead and sternum velocities and range of motions. The mTBI group demonstrated a slower forehead peak angular velocity in all exercises, which may be a compensatory strategy to manage balance issues or symptom exacerbation. Additionally, the mTBI group exhibited a larger forehead range of motion during specific exercises, potentially linked to proprioceptive deficits. These findings emphasize the usefulness of utilizing IMUs to monitor the quality of home-based vestibular exercises for individuals with mTBI and the potential for IMUs improving rehabilitation outcomes.

## 1. Introduction

Each year, approximately 2.5 million individuals in the United States experience a traumatic brain injury (TBI), with about 84% of these cases categorized as mild TBI (mTBI) [1,2]. After mTBI, dizziness and balance problems are frequently reported, with a prevalence ranging from 23% to 81% [3,4]. In fact, immediately after sustaining an mTBI, up to 81% of individuals report experiencing dizziness, and approximately 18% continue to complain of dizziness up to three months post-injury [5,6,7]. More than 40% of those with vestibular-related dizziness are unable to work and 19% are restricted to their homes [8]. The persistent presence of balance and dizziness problems has a substantial impact on anxiety levels and reintegration into the workforce, and may help explain why individuals with a recent history of mTBI face a three-fold higher risk of incurring a second mTBI and an increased risk of musculoskeletal injuries [9,10,11,12,13].

Vestibular rehabilitation is a common method for treating dizziness and balance issues in various populations. This approach involves instructing patients to engage in specific exercises that rely on precise head movements and balance activities [5,14,15,16]. The goal of vestibular rehabilitation is to enhance gaze stability and postural control. There is weak, yet promising, evidence supporting the effectiveness of vestibular rehabilitation after mTBI [17,18]. However, the studies are small and lack standardization. Therefore, rehabilitation efforts for regaining balance and reducing dizziness in individuals with mTBI remain a challenging task [19,20]. Consequently, there exists a need for larger studies to conclusively establish the efficacy of such rehabilitation for mTBI. Furthermore, it is worth noting that subthreshold exercise may alleviate symptoms, but does not appear to reduce the time to recovery, and standalone therapies including vestibular and ocular-motor exercises have limited supporting evidence for yielding improvements [21].

One possible reason for less-than-optimal rehabilitation outcomes may stem from poor performance in vestibular rehabilitation exercises. As vestibular rehabilitation is most often performed at home without physical therapist supervision, factors such as speed, range of motion (ROM), and substitution patterns in these exercises are not monitored. Individuals with vestibular issues often have a compromised perception of head position and movement, which may lead them to perform an exercise with an altered ROM [22,23,24]. The presence of mTBI symptoms may limit the ability of a person to rotate their head quickly [25]. Moreover, individuals with mTBI tend to exhibit reduced head rotational velocity when instructed to perform activities such as walking with head turns, which is a common daily task [26]. Effective vestibular and balance rehabilitation typically involves progressively increasing the amplitude (i.e., ROM) and velocity of head movements during static and dynamic balance exercises, all while maintaining a stable trunk posture [27,28]. Individuals may compensate for subtle deficits by either restricting head movement or gross movement performance. For example, if fast head turns provoke dizziness and imbalance while performing a complex motor task, then the person may slow down the motor task (i.e., running or walking) or slow down the head movement. This tradeoff may have implications for returning to work, sports, or everyday activities that demand both balance and head turns simultaneously. Key factors in exercise performance, such as the speed and ROM of head movements, may have the potential to influence outcomes [29]. Our understanding of exercise performance in people with mTBI currently remains limited. Notably, a clinical practice guideline for people with peripheral vestibular hypofunction indicated a substantial lack of evidence concerning exercise performance recommendations, and the guideline recommended exploring how variables like exercise speed and ROM impact rehabilitation outcomes [28]. This gap in clinical care is particularly evident, and the velocity of head movement is likely a critical aspect of vestibular rehabilitation for mTBI, but remains poorly defined and challenging to quantify [29].

Inertial measurement units (IMUs) have the potential to aid in quantifying exercise performance at home. The validity of IMU quantification of balance and gait has been demonstrated across various populations with balance issues, including head and trunk movements common in vestibular rehabilitation exercises [30,31,32,33,34,35]. The presence of IMUs in clinical practice offers an alternative, objective, technological solution for assessing head movements, both within clinical environments and in home and community settings [36]. These portable sensors can non-invasively measure head movements specific to exercise tasks aimed at enhancing gaze stability [37,38]. This approach could facilitate the comparison of various movement tasks and can track the progression of vestibular rehabilitation over time. However, while IMUs are common in research, they are not yet used in clinical practice. Therefore, the aim of this study was to quantify vestibular exercise performance at home in people with mTBI using IMUs. We hypothesized that individuals with mTBI would exhibit reduced head ROM and velocity during vestibular exercises, and that IMU measurements would identify poor performance in some participants. Additionally, we hypothesized that individuals with more severe symptoms would perform worse across all vestibular rehabilitation exercises.

## 2. Materials and Methods

### 2.1. Participants

A convenience sample of 50 healthy controls and 73 people with mTBI were recruited (Table 1) as part of a large clinical trial (ClinicalTrials.gov identifier: NCT03479541) [39]. Participants were eligible for this study if they met the following criteria: (1) aged between 18 and 60 years, and (2) displayed minimal to no cognitive impairment (<9 on the Short Blessed Test). Additionally, individuals with mTBI were included if they had received an mTBI diagnosis in accordance with the Veterans Affairs/Department of Defense criteria [1] and were still reporting symptoms related to their mTBI. The exclusion criteria encompassed: (1) the presence of any musculoskeletal, neurological, or sensory issues that could account for balance deficits (excluding mTBI); (2) a moderate-to-severe substance use disorder within the last month; (3) experiencing significant pain during the evaluation (rated as >7/10 based on patient self-report); (4) pregnancy; and (5) the inability to refrain from taking medications that might impair balance for a 24 h period before testing.

### 2.2. Procedure

Each of the participants (healthy controls and people with mTBI) were instructed by a physical therapist in four commonly prescribed vestibular exercises [40,41], which included: (1) gaze stabilization, (2) visual motion sensitivity (VMS), (3) standing balance with head turns and a soft gaze (i.e., relaxing their eyes and without fixating on a single point), and (4) walking while incorporating head turns and maintaining a soft gaze. For the gaze stabilization exercise, participants sat in a chair with their feet shoulder-width apart, holding a popsicle stick with a large ‘A’ letter (size 14 font) at arm’s length. Their goal was to rapidly move their head left and right or up and down over a 60-degree peak-to-peak ROM (30 degrees left and right or 30 degrees up and down) while focusing on the ‘A’ and keeping their trunk still. During the VMS exercise, participants sat in a chair with their feet apart and held the same popsicle stick with both hands at arm’s length. They then either rotated from left to right (~180 degrees peak to peak) or bent up and down (~180 degrees peak to peak), ensuring their trunk moved with their head while keeping the ‘A’ in focus. Their goal was to perform this exercise with a fast speed and with maximum ROM while maintaining focus on the ‘A’. During the standing balance with head turns exercise, participants stood with their feet apart on a firm surface and with their eyes open. They were directed to move their head from left to right or up and down, maximizing speed and ROM while maintaining balance. For the walking with head turns exercise, participants were asked to walk at a self-selected comfortable pace while moving their head from left to right or up and down, maximizing speed and ROM while staying balanced during walking. All exercises were performed two times for 30 s with head motion in both transverse (right/left) and sagittal (up/down) planes. Participants from both groups were instructed to achieve the targeted ROM and velocity while ensuring exercise quality.

Participants wore two IMUs (APDM, a Clario Company, Portland, OR, USA), one attached to the center of the forehead and one to the sternum with elastic straps for each exercise. Each IMU was equipped with a tri-axial accelerometer (with a range of ±6 g), gyroscope (with a range of ±2000 °/s), and magnetometer (with a range of ±6 gauss), and sampled data at 128 Hz. The IMUs employed wireless synchronization to guarantee that multiple units collected data with a high level of accuracy. Data from the two IMUs were transmitted wirelessly to a data communication hub, which was attached to a laptop that was running Moveo (APDM, a Clario Company, Portland, OR, USA) for data collection. For each vestibular exercise, measurements were taken for forehead and sternum ROM, as well as peak angular velocity, in both the transverse and sagittal planes. Previous work has indicated that these IMUs are valid and exhibit moderate-to-excellent reliability to quantify peak angular velocity and ROM from these exercises [32,35].

Information from healthy control participants was gathered within our research facility to establish healthy normative performance while in the presence of physical therapists. The physical therapist ensured the healthy control participants applied the sensors in the proper location and performed all exercises correctly.

Individuals with mTBI were similarly instructed in our research space by a physical therapist at the mTBI participant’s first of eight in-person rehabilitation visits as part of their rehabilitation program for the larger clinical trial. In the first rehabilitation session, the mTBI participants were provided with verbal and written instructions on how to conduct the exercises, properly wear the sensors, and set up the equipment for data collection during the home exercises. During that first session, participants with mTBI also demonstrated competence to the physical therapist on how to wear the sensors, set up the data collection system, and perform the home exercises while wearing the sensors. Afterward, participants with mTBI were provided with the IMU sensors and data collection equipment and directed to perform the exercises in their own home daily over a six-week period on days when they did not attend an in-person visit with the physical therapist. This effectively replicated a home-based exercise program which is standard care.

All participants (healthy control and mTBI) performed each vestibular exercise with the IMU sensors twice in each direction for 30 s. Participants with mTBI were instructed to allow their baseline symptoms to increase by no more than two points above their pre-exercise baseline on a 0–10 scale. If their symptoms increased, they had to reduce head ROM or speed. They were also encouraged to take breaks if any of the exercises triggered symptoms. For the purpose of this paper, we only included the first day of home exercises for analysis. During one of the in-person physical therapy appointments, the participants with mTBI were asked to rate how difficult it was to learn/use the system on a scale of 0 (easy) to 10 (difficult).

### 2.3. Outcomes

Forehead and sternum IMU data were processed with Moveo. Data processing for calculating the peak angular velocity and ROM have previously been described elsewhere [35]. Briefly, forehead and sternum segment positions and orientations were determined from a 3 s still period with no movement prior to exercise performance [35]. Angular velocities were extracted from the forehead and sternum IMUs in the transverse plane for right/left exercises and in the sagittal plane for up/down exercises. Time series data from a 30 s exercise were segmented into individual head turns and allowed for the calculation of the peak angular velocity and ROM of each head turn. Each individual forehead and sternum peak angular velocity and ROM was averaged across each 30 s exercise trial and across the two trials for statistical analysis.

The severity of mTBI participants’ symptoms was assessed using the Neurobehavioral Symptom Inventory (NSI), a self-reported questionnaire that quantifies overall mTBI symptoms experienced during the previous two weeks [42]. The NSI consists of 22 questions, each rated on a scale from 0 (none) to 4 (very severe), resulting in a total score ranging from 0 to 88. This assessment tool has demonstrated good internal consistency and reliability [42,43].

The Dizziness Handicap Inventory (DHI), a recommended measure for mTBI, was used to assess self-perceived handicap due to dizziness within the mTBI group [44]. It consists of 25 questions rated as never (0 points), sometimes (2 points), or always (4 points). The total score, out of 100 points, reflects the level of self-perceived handicap, with higher scores indicating greater impact. The DHI demonstrates excellent test–retest reliability in vestibular populations [45] and is a reliable tool for tracking post-mTBI improvement following vestibular rehabilitation [46]. Both the NSI and DHI were collected during laboratory visits as part of the larger clinical trial approximately one week before mTBI participants started vestibular home exercises with the IMUs.

### 2.4. Statistics

To quantify the vestibular therapy home exercise performance in people with mTBI, we compared the forehead and sternum peak angular velocities and ROM in people with mTBI to healthy controls with parametric (independent Welch’s *t*-tests) and non-parametric (Mann–Whitney U or bootstrapped Fligner–Policello robust rank order test) tests [47,48], depending on normality. To help interpret the impaired velocity and ROM in people with mTBI, we further examined the forehead peak angular velocity exercise performance relationships within participants and with symptoms. Specifically, we ran Pearson linear correlations on the forehead peak angular velocity from each exercise and direction. Additionally, we used Pearson linear correlations to examine the forehead peak angular velocities from the vestibular exercises and their relationships with symptoms (NSI and DHI total scores). All significance values (group comparisons and correlations) were adjusted for multiple comparisons using a Benjamini–Hochberg false discovery rate correction [49] and were considered significant at an adjusted *p* < 0.05. Statistical tests were performed in MATLAB (Version 2022b).

## 3. Results

The participant demographics for the mTBI and healthy control groups are presented in Table 1. There was no difference in age between the mTBI group and the healthy control group (*p* = 0.082). Just under half of the mTBI group received their mTBI from a motor vehicle accident and just over half self-reported no previous mTBI. On average, the mTBI group was 74 days removed from their initial injury when starting vestibular rehabilitation. Participants were still symptomatic from their injuries (Table 1). The mTBI participants found the system easy to learn and use (median = 1; range = 0, 10) when asked how difficult it was to learn/use the IMU sensor system during vestibular home exercises.

### 3.1. The mTBI Group Demonstrated Significantly Slower Forehead Velocity across All Exercises and Directions

The mTBI group had a significantly slower forehead peak angular velocity compared to the healthy control group across all exercises and both directions (Table 2; adjusted *p*’s < 0.01). The largest difference between the mTBI and healthy control group was in the standing balance with horizontal head turns exercise (mean difference (lower and upper 95% confidence interval = −146.8 °/s (−185.0 °/s, −108.6 °/s)) and the smallest difference was in the vertical gaze stabilization exercise (−34.5 °/s (−55.8 °/s, −13.2 °/s)). The following paragraphs describe the group differences on forehead ROM, sternum ROM, and sternum peak angular velocities according to each exercise.

### 3.2. Gaze Stabilization

In addition to the slower forehead peak angular velocity, the mTBI group had a significantly larger forehead ROM compared to the healthy control group for the horizontal and vertical directions (Table 2; adjusted *p*’s < 0.001). The mTBI group had a significantly larger sternum ROM compared to the healthy control group in the horizontal direction (Table 2; adjusted *p* < 0.001), but there was no difference between groups for sternum ROM in the vertical direction (Table 2; adjusted *p* = 0.060). There were no group differences for sternum peak angular velocity (Table 2; adjusted *p*’s > 0.093).

### 3.3. Visual Motion Sensitivity (VMS)

In addition to the slower forehead peak angular velocity, the mTBI group had significantly slower sternum peak angular velocities compared to the healthy control group for both the horizontal and vertical directions (Table 2, adjusted *p*’s < 0.001). The mTBI group had a significantly smaller sternum ROM compared to the healthy control group, but only in the vertical direction (Table 2; adjusted *p* = 0.005). There were no group differences in forehead ROM across horizontal and vertical visual motion sensitivity exercises (Table 2; adjusted *p*’s > 0.059).

### 3.4. Standing Balance with Head Turns

The mTBI group demonstrated a significantly slower sternum velocity compared to the healthy control group for the horizontal (adjusted *p* = 0.005) and vertical directions (adjusted *p* = 0.005). There were no other group differences across directions for forehead and sternum ROM (Table 2; adjusted *p*’s > 0.154).

### 3.5. Walking with Head Turns

The mTBI group demonstrated a slower forehead peak angular velocity. There were no significant differences between groups for forehead ROM, sternum ROM, and sternum peak angular velocities across directions for the walking with head turns exercise (Table 2; adjusted *p*’s > 0.060).

### 3.6. People Who Performed Worse (Slower Peak Angular Forehead Velocity) in One Vestibular Home Exercise Were the Same People Who Performed Worse across All Other Exercises

There were significant correlations of forehead peak angular velocity among all the vestibular exercises and directions (adjusted *p*’s < 0.004). Specifically, participants with slow forehead peak angular velocities in an exercise generally had slow forehead peak angular velocities on other exercises (Pearson correlation coefficients ranged from 0.38 to 0.87; Table 3).

### 3.7. No Relationships Existed between Forehead Peak Angular Velocities from Vestibular Therapy Home Exercises with mTBI Symptoms

Overall, the symptoms reported approximately one week before home exercises on the NSI and DHI did not significantly correlate with forehead peak angular velocities on any of the vestibular therapy home exercises within the mTBI group (adjusted *p*’s > 0.053; Table 3). The exception was that lower NSI total scores significantly correlated with faster peak angular forehead velocities on vertical gaze stabilization exercises and the strength of this relationship was considered small (Table 3; adjusted *p* = 0.015).

## 4. Discussion

In this study, we found significant differences in the performance of a vestibular home exercise program between individuals with mTBI and healthy controls when quantified with IMUs. Specifically, participants with mTBI exhibited a slower forehead peak angular velocity in all four exercises, both in the transverse and sagittal planes. Additionally, we identified other patterns indicative of poor exercise quality and performance during home-based vestibular exercises. For example, the mTBI group demonstrated a larger forehead ROM during some exercises and an inability to stabilize the trunk in other exercises.

Our results supported one of our hypotheses, showing that people with mTBI performed all exercises with a slower forehead velocity than healthy controls. Previous studies suggest that there is a change in the central integration of balance sensory systems after mTBI, which results in a deficit in balance control [50]. Participants with mTBI may slow their head movements to compensate for balance disturbances during the exercises. These changes may be related to a drive to preserve stability and offset the deficits in postural control. This slowing to preserve stability is in line with other studies that show a decrease in gait speed and turning speed after mTBI [25,51]. Another explanation for the decreased head velocity across exercises could be related to symptom provocation. Dizziness is a commonly reported symptom after mTBI and head movements may exacerbate symptoms [5,52]. Due to previous research showing a correlation between dizziness and postural sway deficits in individuals with mTBI, participants may perform head turns more slowly, reflecting their baseline experience of dizziness [50]. However, most of our correlations between laboratory assessments of symptoms (obtained approximately one week before starting home exercises) and velocity performance were not significant.

One important part of both gaze stabilization and VMS exercise is to keep the target in focus. There is evidence to suggest that after mTBI, both ocular-motor and vestibular deficits are present and contribute to balance impairments [53,54]. Immediately acutely following mTBI, ocular-motor deficits may occur in up to 90% of individuals and peripheral vestibular deficits in 14–90% of patients [55]. Problems with gaze stabilization and ocular-motor function may have altered the participants’ ability to focus on the target and prevented participants with mTBI from being able to perform the exercises at a velocity similar to the healthy controls. Importantly though, the slowing of head velocity is problematic because there is evidence to suggest that there is a minimum speed at which vestibular exercises need to be performed to reap exercise benefits [29]. It is possible that participants with mTBI cannot perform exercises at a higher speed and may need more time and practice to build up to a higher velocity consistent with healthy people.

An unexpected finding of this study that did not support our hypothesis was that people with mTBI showed a larger, not smaller, head ROM during vertical and horizontal gaze stabilization than healthy controls. Both groups of participants were instructed to perform gaze stabilization exercises with 30 degrees of head turn in each direction (60 degrees of motion total). One explanation for the larger than expected ROM in people with mTBI could possibly be due to impaired proprioception. Specifically, people with mTBI have been shown to have a decrease in their cervical proprioception, as shown by the Joint Position Error Test [22]. Differences in cervical proprioception can cause people with mTBI to both overshoot and undershoot a target. This could cause people with mTBI to have a greater than intended ROM during an exercise with a target range (vs. the other exercises which instruct the patient to turn their head as much as possible and pose a physical limitation vs. a target range). Alternatively, it could be due to unintended deviation from directions for the home exercise program, since these exercises were performed at home without supervision. However, we have no way to verify this, since people were unsupervised.

**Figure 1:**
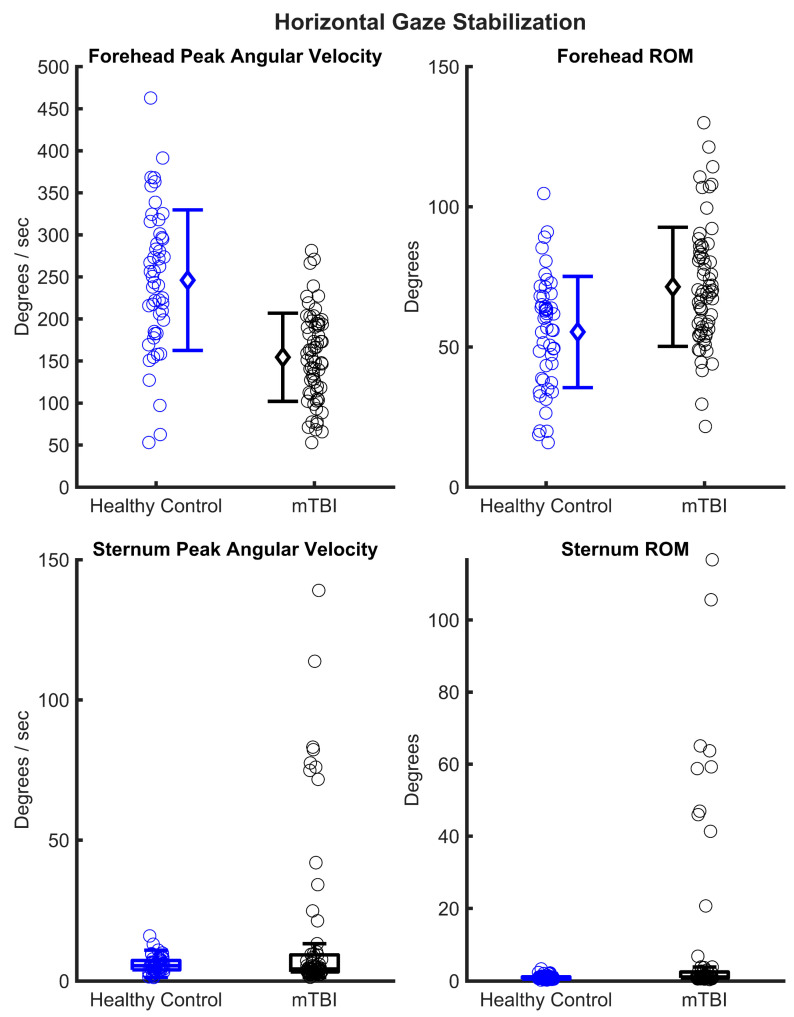
Individual participant data for the healthy control (blue) and mild traumatic brain injury (mTBI) group (black) for horizonal gaze stabilization exercise performance (forehead and sternum peak angular velocities and range of motion (ROM)). In the forehead peak angular velocity and ROM figures, mean ± standard deviations are indicated by open diamond. In the sternum peak angular velocity and ROM figures, box and whiskers are presented.

In several exercises, the role of trunk motion differs. During gaze stabilization, the sternum is meant to stay still, while the head moves. However, the mTBI group showed a larger trunk motion compared to healthy controls. One explanation for this could be that people with mTBI may be attempting to minimize head-on-trunk motion due to symptom exacerbation. For the VMS exercise, in contrast to gaze stabilization, the head and trunk are meant to rotate together. Here, people with mTBI showed a reduced trunk ROM during vertical VMS and a reduced trunk peak angular velocity during standing balance with head turns and vertical and horizontal VMS. These changes in trunk motion suggest that participants may be limiting body motion to reduce symptom exacerbation or balance perturbation. An alternative explanation to the large ROM observed in gaze stabilization exercises could be from the mTBI participants performing the exercise incorrectly. Figure 1, for example, shows the mTBI participants with an abnormally fast sternum peak velocity and large sternum ROM. It is possible that these mTBI participants, specifically those with sternum peak angular velocities greater than 50 deg/s or those with a sternum ROM greater than 40 degrees, are performing the wrong exercise and could be performing a VMS-type exercise. However, this remains speculative, as we were not present while the mTBI participants performed these exercises at home. Nonetheless, observing these data as a clinician may prompt further investigation with a patient on if they are performing home exercises correctly.

The majority of mTBI participants found the sensors easy to set up to use for vestibular home exercises and were able to successfully record data. This is an important consideration for widespread implementation for tracking vestibular home exercise performance. From a practical perspective, clinicians will likely have an in-person visit with their patients to assess functional limitations and provide instructions for a home exercise program. In our study, the physical therapists demonstrated proper exercise performance and additionally provided proper sensor use and data collection setup for using the sensors at home. Ideal systems that use wearable internal sensors should be user-friendly and easy to administer, which the majority of mTBI participants in our study experienced. Utilizing sensors could improve home exercise compliance during vestibular rehabilitation, which is a known limitation in research studies, as well as clinical practice [56].

There were limitations to this study that should be addressed. The exercises were performed by the mTBI participants in their homes without supervision, thereby limiting our interpretation of some of the findings. It is impossible to know if they were performing the exercises as intended or if they deviated from the given directions (e.g., mismatched directions of moving head as much as possible during gaze stabilization rather than for head turns). This is opposed to the healthy control data, which were all collected under supervision from a physical therapist who could ascertain that the correct directions were followed. Nonetheless, this work shows the utility of using IMU sensors to monitor exercise performance at home. Additionally, we did not record symptoms during or after exercise performance. Therefore, it is unclear if the differences in performance between groups was in part due to pre-exercise symptoms or symptom provocation during the exercise. We also did not track if the healthy control participants had any symptom provocation, so it is unclear if some level of dizziness or headache could be considered normal with these exercises. It is known that peripheral vestibular function decreases as we age, and approximately 49% of people over the age of 60 demonstrate some sort of vestibular dysfunction [57]. Therefore, our results for vestibular home exercise performance (forehead peak angular velocity and ROM) with wearable sensors in both healthy controls and people with mTBI are specific to the current study cohorts and may not be generalizable to older populations or other neurological conditions. Future studies could include a more robust normative data sample that has more participants and participants older than 60 years old. Finally, we did not assess differences in postural sway and/or gait measures between the groups while performing the standing balance and walking exercises. Whereas the gaze stabilization and VMS exercises were performed seated, the head-turning standing balance and walking exercises did involve a component of balance. Therefore, we were unable to assess the relationship between changes in the forehead peak angular velocity and ROM and changes in postural control or gait.

In conclusion, our findings illustrate how individuals with mTBI perform their home exercise programs, which begins to elucidate the potential utility of using IMUs to monitor vestibular exercise performance at home. Since exercise quality is a critical factor for improved efficacy, future efforts should be directed toward monitoring and enhancing exercise quality during home-based programs to track changes over time. Furthermore, a robust normative database would facilitate interpretation. The next step is to explore whether IMU technology can provide real-time feedback to individuals with mTBI during their exercises. Such biofeedback derived from IMUs may expedite these individuals’ progress toward achieving performance levels on par with healthy controls.

## Figures and Tables

**Table 1:** Participant demographics (mean and standard deviation unless noted) for the mild traumatic brain injury (mTBI) group and healthy control group.

	mTBI	Healthy Control
	n = 73	n = 50
Age (years)	36.8 (12.5)	40.7 (12.0)
Height (cm)	170.0 (9.9)	173.9 (10.9)
Days from Injury to Starting Vestibular Rehabilitation	73.9 (31.7)	
Number of Previous mTBIs (N and %)		
0	43 (59%)	
1	15 (21%)	
2	7 (10%)	
3 or more	8 (11%)	
Mechanism of Injury (N and %)		
Motor Vehicle Accident	25 (47%)	
Fall	18 (19%)	
Other	14 (17%)	
Sport	12 (14%)	
Bike	4 (7%)	
DHI Total Score	29.5 (17.2)	
NSI Total Score	36.3 (13.4)	

Abbreviations: N, number; mTBIs, mild traumatic brain injuries; DHI, Dizziness Handicap Inventory; NSI, Neurobehavioral Symptom Inventory.

**Table 2:** Objective measures (means and standard deviations) for forehead and sternum ROM and peak angular velocities from vestibular therapy home exercises for the healthy control (HC) group and the mild traumatic brain injury (mTBI) group.

		Forehead ROM (°)		Forehead Peak Angular Velocity (°/s)		Sternum ROM (°)		Sternum Peak Angular Velocity (°/s)	
		HC	mTBI	HC	mTBI	HC	mTBI	HC	mTBI
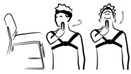 Gaze Stabilization	H	***55 (20)***	***71 (22) ^T^***	***246 (84)***	***159 (52) ^T^***	***1 (1)***	***12 (26) ^FP^***	6 (3)	18 (31)
	V	***37 (16)***	***49 (15) ^T^***	***169 (60)***	***138 (56) ^U^***	2 (1)	2 (1)	12 (6)	10 (6)
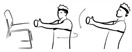 Visual Motion Sensitivity	H	115 (26)	112 (32)	***197 (56)***	***140 (50) ^U^***	104 (23)	99 (32)	***180 (51)***	***123 (45) ^U^***
	V	103 (27)	91 (32)	***163 (46)***	***116 (41) ^T^***	***68 (28)***	***50 (28) ^U^***	***118 (42)***	***67 (30) ^FP^***
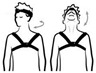 Standing Balance with Head Turns	H	121 (22)	118 (22)	***414 (106)***	***272 (98) ^T^***	8 (8)	7 (5)	***23 (15)***	***16 (11) ^FP^***
	V	78 (20)	80 (23)	***266 (77)***	***188 (75) ^T^***	6 (2)	5 (3)	***27 (10)***	***20 (13) ^FP^***
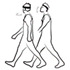 Walking with Head Turns	H	123 (27)	127 (29)	***386 (80)***	***294 (91) ^U^***	29 (14)	37 (18)	67 (24)	69 (19)
	V	73 (20)	76 (21)	***257 (64)***	***201 (72) ^U^***	8 (2)	9 (3)	47 (12)	45 (15)

Bold and italicized rows indicate a significant difference between the HC and the mTBI groups (Benjamini–Hochberg adjusted *p* < 0.05). Superscripts denotes the statistical test: ^T^, Welch’s *t*-test; ^U^, Mann–Whitney U test; ^FP^, bootstrapped Fligner–Policello robust rank order test. Abbreviations: ROM, range of motion; HC, healthy control; mTBI, mild traumatic brain injury; H, horizontal; V, vertical.

**Table 3:** Forehead peak angular velocity relationships (Pearson linear correlation coefficients) between vestibular home exercises and relationships with Neurobehavioral Symptom Inventory (NSI) and Dizziness Handicap Inventory (DHI) total scores within the mild traumatic brain injury (mTBI) group.

	Gaze Stabilization		Visual Motion Sensitivity		Standing Balance with Head Turns		Walking with Head Turns	
	H	V	H	V	H	V	H	V
Gaze Stabilization (V)	***0.81***							
Visual Motion Sensitivity (H)	***0.68***	***0.64***						
Visual Motion Sensitivity (V)	***0.57***	***0.59***	***0.77***					
Standing Balance with Head Turns (H)	***0.66***	***0.66***	***0.59***	***0.57***				
Standing Balance with Head Turns (V)	***0.60***	***0.68***	***0.62***	***0.59***	***0.87***			
Walking with Head Turns (H)	***0.63***	***0.55***	***0.38***	***0.40***	***0.74***	***0.74***		
Walking with Head Turns (V)	***0.61***	***0.66***	***0.48***	***0.57***	***0.70***	***0.78***	***0.80***	
NSI Total Score	−0.28	***−0.39***	−0.20	−0.06	−0.28	−0.28	−0.25	−0.18
DHI Total	−0.19	*−0.32*	−0.11	−0.17	*−0.32*	−0.28	−0.19	−0.27

Bolded and italicized cells indicate significant Pearson correlation (Benjamini–Hochberg adjusted *p* < 0.05). Abbreviations: mTBI, mild traumatic brain injury; H, horizontal; V, vertical.

## Data Availability

Data will be available to qualified investigators through the Federal Interagency Traumatic Brain Injury Research (FITBIR) Informatics System at https://doi.org/10.23718/FITBIR/1518823, through the National Institutes of Health Center for Information Technology: https://fitbir.nih.gov/content/access-data.
